# A Rout to Protect Quantum Gates constructed via quantum walks from Noises

**DOI:** 10.1038/s41598-018-25550-1

**Published:** 2018-05-08

**Authors:** Yi-Mu Du, Li-Hua Lu, You-Quan Li

**Affiliations:** 1 Department of Physics, Zhejiang University, Hangzhou, 310027 P. R. China; 2 Collaberative Innovation Center of Advanced Microstructure, Nanjing, P. R. China

## Abstract

The continuous-time quantum walk on a one-dimensional graph of odd number of sites with an on-site potential at the center is studied. We show that such a quantum-walk system can construct an *X*-gate of a single qubit as well as a control gate for two qubits, when the potential is much larger than the hopping strength. We investigate the decoherence effect and find that the coherence time can be enhanced by either increasing the number of sites on the graph or the ratio of the potential to the hopping strength, which is expected to motivate the design of the quantum gate with long coherence time. We also suggest several experimental proposals to realize such a system.

## Introduction

The quantum walk, proposed as the quantum mechanical counterpart of the classical random walk^1^, has received increasing attentions in recent years due to its extensive potential application in many fields^2–6^. For example, the quantum walk can offer us a simple model to simulate the quantum information processing^7–9^. In order to realize the quantum information processing, one of the essential issues is to manipulate the quantum state coherently by quantum gates which have been experimentally implemented in some conventional systems, such as Josephson-junction devices^10,11^, nuclear magnetic resonant^12,13^ and trapped ions^14–16^, where the decoherence of the quantum gates was studied^17–19^. As we know, there is a natural mapping between the quantum walk and qubit systems^8^, which implies that the quantum walk has potential applications in the universal quantum computation^2,3^. As we aware, there are several works that studied the universal quantum computation with the help of quantum walks. For example, In refs^2,3,20–24^, the authors presented suggestions to realize different quantum operations based on the continuous-time quantum walk model. Whereas, in ref.^25^, the authors proposed several schemes to realize the universal quantum computation based on the discrete-time quantum walk.

Since the quantum walk has been experimentally realized in quite several systems^26–28^ and its decoherence has been theoretically studied^29^, whether an appropriate quantum-walk system can be used to increase the fidelity of quantum gate is undoubtedly an important issue in the quantum information processing. We will suggest two experimental proposals to realize it and show how to suppress the decoherence effect for certain quantum gates.

In this paper, we start from a study on a continuous-time quantum walk on a one-dimensional graph of odd number of sites with an on-site potential at the central site. As there are several schemes to realize such a quantum-walk model in experiment, such as coupled superconductor LC circuits bridged by a microwave resonator, or voltaged quantum dot array, we show that the continuous-time quantum walk can be applied to construct different type of quantum gates, and the type of which depends on the initial state and the number of the sites on the graph. We further study the decoherence effect of the quantum gate constructed by the continuous-time quantum walk and show that such a decoherence effect can be suppressed by appropriately tuning the parameters of the quantum-walk system or increasing the number of sites on the graph, which enlightens one to optimize the capability of the quantum gate, such as the suppress of decoherence, the enhancement of fidelity in the quantum operation.

This paper is organized as follows. In the next section, we give the model of the continuous-time quantum walk of a single particle and suggest two experimental proposals to realize the model we considered. In sec. 1.1, we take the simplest graph of three sites as an example to study the construction of an X-gate for a single qubit as well as a control gate for two qubits. We also study the decoherence properties of the constructed quantum gates. In sec. 1.2 and 1.3, we investigate the graph of five sites and any odd number of sites. In sec. 2, we discuss the construction of control gate. At last, we give a brief summary.

We consider the continuous-time quantum walk of a single particle on the following one-dimensional graph.

The graph consists of 2*n* + 1 sites, and the on-site potential at the central site differs from that at the other sites. For such a single-particle system, the Hilbert space is spanned by a set of orthogonal bases {|*j*〉, |*j* = −*n*, −*n* + 1, −*n* + 2, …, *n* − 1, *n*}, where |*j*〉 denotes the state that a particle occupies the *j*th site on the graph. The graph implies that the tunnelling occurs between the nearest-neighbour sites, then the Hamiltonian is given by

1 $$H=\sum _{j=-n}^{n-1}\,J(|j\rangle \langle j+1|+{\rm{H}}.\,{\rm{c}}.\,)+ {\mathcal E} |0\rangle \langle 0|,$$

where *J* refers to the hopping strength and ${\mathcal E}$ to the on-site potential at the central site. The importance of the study on such a model is that can be realized by several systems in experiment.

**Figure 1:**
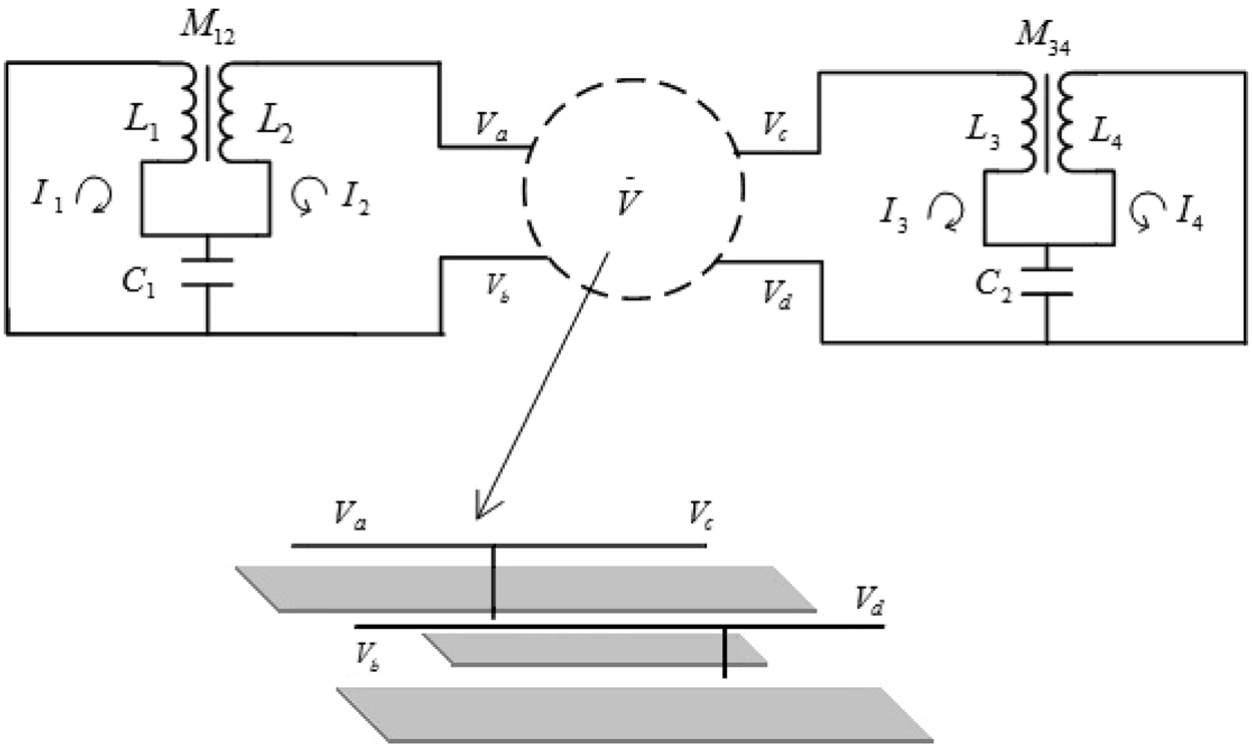
The sketch of two coupled superconductor LC circuits bridged by a microwave resonator where *C*’s, *L*’s and *M*’s refer to the capacitance, self- and mutual-inductance, respectively. Such a setup can simulate the quantum walk on the graph of five sites.

One proposal to realize the model (1) is two series of superconductor LC circuits^30,31^ bridged by a microwave resonator. That is, *n* LC circuits couple to each other, of which one terminal is connected to a microwave resonator, and the resonator furthermore connects to another *n* coupled LC circuits. The resonator bridging those two LC-circuit series plays the role of an AC voltage source of single mode to each terminals. The second quantization version of such a system can be modelled by our model Hamiltonian (1) as long as the single-photon process is considered. As an example, Fig. 1 shows the scheme of the setup with *n* = 2 that can simulate the quantum walk on the one-dimensional graph of five sites.

**Figure 2:**
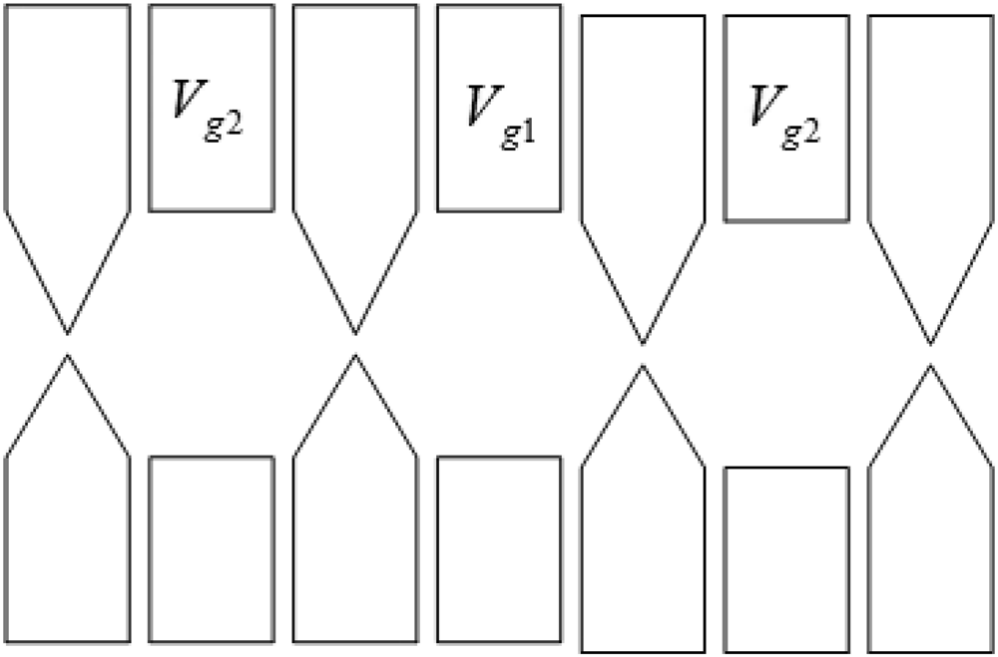
The sketch of the quantum dot array to realize the model (1). The ${\mathcal E}$ can be realized by tuning the voltage *V*_*g*1_ and *V*_*g*2_.

Another proposal to realize the model (1) can be a quantum dot array that was implemented in many experiments^32,33^. We show the sketch of the quantum dot array for *n* = 1 as an example in Fig. 2. The on-site potential ${\mathcal E}$ in our model can be produced either by applying different voltages *V*_*g*1_ and *V*_*g*2_^33^ or by placing a STM tip focusing on the central quantum dot.

## Results

### The construction of quantum gates for a single qubit

In order to construct quantum gates for a single qubit, it will be more illustrative to express (1) as follows

2 $$H=\sum _{l,\sigma }\,J(|l+1,\sigma \rangle \langle l,\sigma |+{\rm{H}}.\,{\rm{c}}.\,)+ {\mathcal E} |0\rangle \langle 0|+J(|1,-\rangle \langle 0|+|0\rangle \langle 1,+\,|+\,{\rm{H}}.\,{\rm{c}}.\,),$$

where the summation is taken over *σ* = ± and *l* = 1, 2, …, *n* − 1. This manifests that the Hilbert space has the following decomposition ${{\mathbb{H}}}_{1}\otimes {{\mathbb{H}}}_{2}\oplus {{\mathbb{H}}}_{3}$. Here ${{\mathbb{H}}}_{1}$ is spanned by {|*l*〉 |*l* = 1, 2, …, *n*}, ${{\mathbb{H}}}_{2}$ by {|+〉, |−〉}, and ${{\mathbb{H}}}_{3}$ by |0〉. We know that one only need two bases to describe a single-qubit system, so we need reduce the total Hilbert space into two dimensional subspace. In order to achieve this goal, we take partial trace over the index *l*. And for convenience of taking partial trace, we write the Hilbert space of the system in the form of ${{\mathbb{H}}}_{1}\otimes {{\mathbb{H}}}_{2}\oplus {{\mathbb{H}}}_{3}$. Here the Hilbert subspace ${{\mathbb{H}}}_{3}$ corresponds to the central site on the graph. Its existence ensures our system consisting of more than 3 sites (i.e., *n* > 1) can always be used to construct a quantum gate for a single qubit.

Now we are in the position to construct a quantum gate for a single qubit and study the relevant decoherence properties. We will investigate the simplest graph of three sites and the graph of five sites successively. Then we generalize our study to the case of any odd number of sites.

#### The case of three-site graph

The simplest case is the graph of three sites for which the Hilbert space is three dimensional. The eigenenergies are solved as 0, *E*_−_/2 and *E*_+_/2 where ${E}_{-}= {\mathcal E} -\sqrt{{ {\mathcal E} }^{2}+8{J}^{2}}$, ${E}_{+}= {\mathcal E} +\sqrt{{ {\mathcal E} }^{2}+8{J}^{2}}$. The corresponding eigenstates can be easily obtained by the standard procedure of linear algebra. In the case of ${\mathcal E} \gg J$, the eigenenergies become 0, $-\,2{J}^{2}/ {\mathcal E}$ and ${\mathcal E} +2{J}^{2}/ {\mathcal E}$, and the corresponding eigenstates are approximately given by

3 $$\begin{array}{rcl}|{\phi }_{1}\rangle  & = & \frac{1}{\sqrt{2}}(|1,+\,\rangle -|1,-\,\rangle ),\\ |{\phi }_{2}\rangle  & = & \frac{1}{\sqrt{2}}(|1,+\,\rangle +|1,-\,\rangle ),\\ |{\phi }_{3}\rangle  & = & |0\rangle .\end{array}$$

Clearly, the eigenenergies for the states |*φ*_1_〉 and |*φ*_2_〉 are closer to each other, while they are much lower than the third eigenenergy for the state |*φ*_3_〉 as long as ${\mathcal E} \gg J$. Therefore these two states provide us an effective two-level system that plays the role of a qubit. The effective Hamiltonian then turns to

4 $${H}_{{\rm{eff}}}=-\,\frac{{J}^{2}}{ {\mathcal E} }{\hat{\sigma }}_{x},$$

where ${\hat{\sigma }}_{x}$ denotes the *x*-component of the Pauli matrix, namely ${\hat{\sigma }}_{x}$ = |+〉 〈−|+|−〉 〈+|.

The time evolution of any given initial state is computable because the above effective Hamiltonian (4) is equivalent to that of a spin-1/2 system in an external magnetic field along *x*-axis with magnitude $2{J}^{2}/ {\mathcal E}$. The expectation values of the Pauli matrices for the solved state define a pseudo-spin polarization vector,

$$\begin{array}{rcl}{S}_{x}(t) & = & \cos (\theta ),\\ {S}_{y}(t) & = & \sin (\theta )\,\cos (-\frac{2{J}^{2}}{ {\mathcal E} }t+\varphi ),\\ {S}_{z}(t) & = & \sin (\theta )\,\sin (-\frac{2{J}^{2}}{ {\mathcal E} }t+\varphi ),\end{array}$$

where *θ* and *ϕ* is determined by the initial state. As the above pseudo-spin polarization vector processes around the *x*-axis periodically, we can have an *X*-gate by choosing an appropriate time duration. This manifests that the continuous-time quantum walk of a single particle on a one-dimensional graph of three sites can construct an *X*-gate for a single qubit.

As is known that the decoherence^34,35^ is inevitable in any realistic system. Now let us introduce noise to the aforementioned system by adding some random fluctuations in the parameters ${\mathcal E}$ and *J*, namely, ${\mathcal E} =\bar{ {\mathcal E} }+\delta  {\mathcal E} (t)$, $J=\bar{J}+\delta J(t)$ where $\bar{ {\mathcal E} }$ and $\bar{J}$ denote their average values, $\delta  {\mathcal E} (t)$ and *δJ*(*t*) the random fluctuations. We assume that the fluctuation of the parameter changes on time slowly enough such that there will be no transitions between the low-lying states {|*φ*_1_〉, |*φ*_2_〉} and the high-lying state |*φ*_3_〉 occurring. The transition between the eigenstates |*φ*_1_〉 and |*φ*_2_〉 also does not occur for their symmetries are different. The adiabatic phase of these two low-lying eigenstates also vanishes due to $\langle {\dot{\phi }}_{\mathrm{1(2)}}|{\phi }_{\mathrm{1(2)}}\rangle =0$ in which the overhead dot denotes the derivative with respect to time.

In such an adiabatic approximation, we can approximately express the effective Hamiltonian as

5 $${H^{\prime} }_{{\rm{eff}}}=(-\frac{{\bar{J}}^{2}}{\bar{ {\mathcal E} }}-\frac{2\bar{J}}{\bar{ {\mathcal E} }}\delta J+\frac{{\bar{J}}^{2}}{{\bar{ {\mathcal E} }}^{2}}\delta  {\mathcal E} ){\hat{\sigma }}_{x},$$

up to the first order of the small quantities owning to the assumption $\delta  {\mathcal E} (t)\ll \bar{ {\mathcal E} }$ and $\delta J(t)\ll \bar{J}$. Then the density matrix at a time *t* can be expressed as $\rho (t)=\exp (-\frac{i}{\hslash }\,{\int }_{0}^{t}\,{H^{\prime} }_{{\rm{eff}}}(t^{\prime} ){\rm{d}}t^{\prime} )\rho (0)\,\exp (\frac{i}{\hslash }\,{\int }_{0}^{t}\,{H^{\prime} }_{{\rm{eff}}}(t^{\prime} ){\rm{d}}t^{\prime} )$, where ${H^{\prime} }_{{\rm{eff}}}$ is given by (5) and *ρ*(0) is determined by the initial state. Since ${\mathcal E}$ and *J* fluctuate on time randomly, we can evaluate the average-density matrix at the time *t* by taking average over ensembles.

6 $${\langle \rho (t)\rangle }_{{\rm{ens}}}=\int \,{\rm{D}}[\delta J]{\rm{D}}[\delta  {\mathcal E} ]\rho (t)\,\exp (-\frac{1}{2}\,\int \,dt\,dt^{\prime} F(t,t^{\prime} )),$$

with $F(t,t^{\prime} )=\delta J(t)A(t-t^{\prime} )\delta J(t^{\prime} )$ + $\delta  {\mathcal E} (t)B(t-t^{\prime} )\delta  {\mathcal E} (t^{\prime} )$ + $\delta  {\mathcal E} (t)C(t-t^{\prime} )\delta J(t^{\prime} )$. Here *A*(*t* − *t*′), *B*(*t* − *t*′) and *C*(*t* − *t*′) are correlation functions characterizing the correlation between the fluctuations, and they are positive and invariant under permutation of *t* and *t*′. If the correlation functions are assumed in the same order, the first term in Eq. (6) (i.e. the term including *A*(*t* − *t*′)) is the leading term due to the assumption ${\mathcal E} \gg J$. Then we can neglect the fluctuation of ${\mathcal E}$ and only consider the decoherence effect caused by the fluctuation of *J*.

In order to characterize the decoherence effect, we evaluate the expectation values of the Pauli matrices at time *t* with the help of ${\langle {S}_{i}\rangle }_{{\rm{ens}}}={\rm{Tr}}({\hat{\sigma }}_{i}{\langle \rho (t)\rangle }_{{\rm{ens}}})$ in which *i* = *x*, *y*, *z*. These values just define a pseudo-spin polarization vector whose module varying on time,

7 $$\begin{array}{rcl}{\langle {S}_{x}\rangle }_{{\rm{ens}}} & = & \cos \,\theta ,\\ {\langle {S}_{y}\rangle }_{{\rm{ens}}} & = & \sin \,\theta \,\cos (-\frac{2{\bar{J}}^{2}}{\bar{ {\mathcal E} }}t+\varphi )\,\exp \,[-4{(\frac{\bar{J}}{\bar{ {\mathcal E} }})}^{2}I(t)],\\ {\langle {S}_{z}\rangle }_{{\rm{ens}}} & = & \sin \,\theta \,\sin (-\frac{2{\bar{J}}^{2}}{\bar{ {\mathcal E} }}t+\varphi )\,\exp [-4{(\frac{\bar{J}}{\bar{ {\mathcal E} }})}^{2}I(t)],\end{array}$$

where $I(t)=\frac{1}{(2\pi )}\,\int \,\frac{{\sin }^{2}(\omega t/2)}{{\omega }^{2}\tilde{A}(\omega )}{\rm{d}}\omega$ with $\tilde{A}(\omega )$ being the Fourier transform of *A*(*t* − *t*′). From the result shown in Eq. (7), we can find that the decoherence effect occurs in the constructed *X*-gate once the fluctuation of parameters is considered. Considering several typical noises with different spectra, such as a constant spectrum and a Gaussian spectrum as well as a Lorentz spectrum, we find that the coherence time is proportional to ${(\bar{ {\mathcal E} }/\bar{J})}^{2}$, which implies that one can enhance the coherence time by increasing the ratio of the parameters $\bar{ {\mathcal E} }$ to $\bar{J}$.

#### The case of five-site graph

Now we turn to the case of the graph of five sites for which the Hilbert space is five dimensional. Just like the case of three-site graph, we are interested in the regime ${\mathcal E} \gg J$. In this case, one eigenvalue ${\mathcal E} +2{J}^{2}/ {\mathcal E}$ is much higher than the other four eigenvalues, *J*, −*J*, $J-{J}^{2}/ {\mathcal E} -{J}^{3}/\mathrm{(2}{ {\mathcal E} }^{2})$ and −$J-{J}^{2}/ {\mathcal E} +{J}^{3}/\mathrm{(2}{ {\mathcal E} }^{2})$. The eigenstates corresponding to those four lower eigenvalues are

8 $$\begin{array}{rcl}|{\phi }_{1}\rangle  & = & \frac{1}{2}(|2,+\,\rangle +|1,+\,\rangle -|1,-\,\rangle -|2,-\,\rangle ),\\ |{\phi }_{2}\rangle  & = & \frac{1}{2}(|2,+\,\rangle -|1,+\,\rangle +|1,-\,\rangle -|2,-\,\rangle ),\\ |{\phi }_{3}\rangle  & = & \frac{1}{2}(|2,+\,\rangle +|1,+\,\rangle +|1,-\,\rangle +|2,-\,\rangle ),\\ |{\phi }_{4}\rangle  & = & \frac{1}{2}(|2,+\,\rangle -|1,+\,\rangle -|1,-\,\rangle +|2,-\,\rangle ).\end{array}$$

They provide an effective four-level system that plays the role of two coupled qubits. By taking a partial trace over the labels *l* = 1, 2, we can obtain a reduced two-level system which constructs a quantum gate for a single qubit. The concrete type of the quantum gate depends on the initial state of the system. Such a quantum gate can suppress the decoherence effect induced by noises for some ideal cases, which will be discussed in the following.

**Figure 3:**
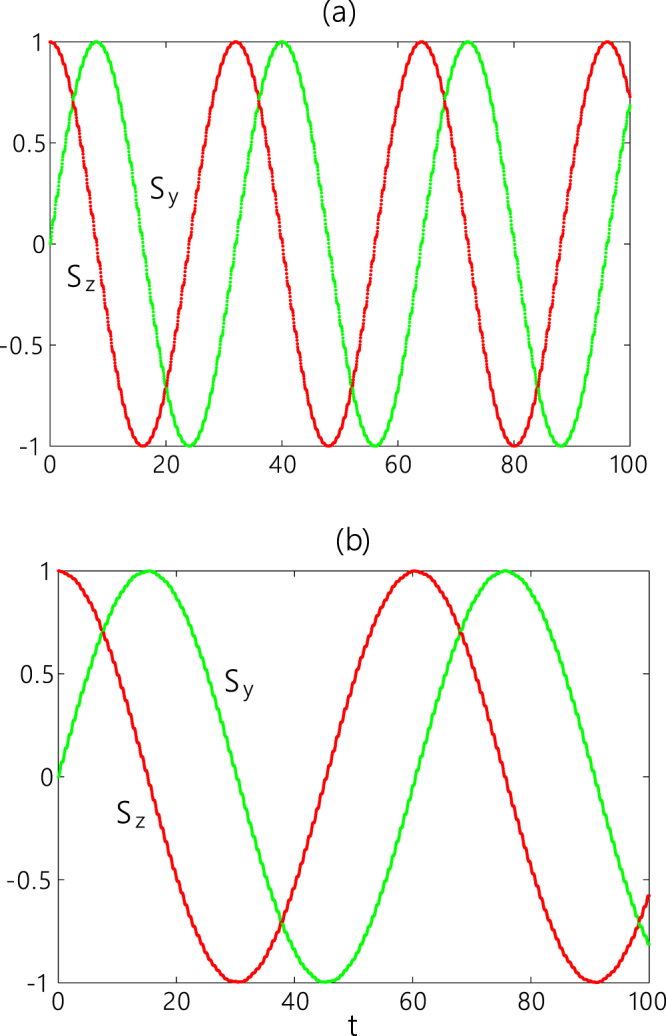
The time evolution of the *y*- and *z*-components of the pseudo-spin polarization vector for the graph of three sites (**a**) and that of five sites (**b**) with parameter choices *J* = 1 and ${\mathcal E} =10$. The initial states are |1, −〉 in (**a**) and $(|2,-\,\rangle +|1,-\,\rangle )/\sqrt{2}$ in (**b**).

We consider an initial state $|\psi \mathrm{(0)}\rangle =\,\cos (\alpha /2\mathrm{)|2},-\,\rangle +\,\sin (\alpha /2){e}^{i\beta }\mathrm{|1},-\,\rangle$ which corresponds to a pseudo-spin polarization vector *S*_*x*_(0) = *S*_*y*_(0) = 0, *S*_*z*_(0) = 1 by taking a partial trace over the labels *l* = 1, 2. We can evaluate the value of the pseudo-spin polarization vector at the time *t*,

9 $$\begin{array}{rcl}{S}_{x}(t) & = & 0,\\ {S}_{y}(t) & = & \sin (\frac{{J}^{2}}{ {\mathcal E} }t)\,\cos (\frac{{J}^{3}}{2{ {\mathcal E} }^{2}}t)\\  &  & +\,\cos \,\beta \,\sin \,\alpha \,\cos (\frac{{J}^{2}}{ {\mathcal E} }t)\,\sin (\frac{{J}^{3}}{2{ {\mathcal E} }^{2}}t),\\ {S}_{z}(t) & = & \cos (\frac{{J}^{2}}{ {\mathcal E} }t)\,\cos (\frac{{J}^{3}}{2{ {\mathcal E} }^{2}}t)\\  &  & -\,\cos \,\beta \,\sin \,\alpha \,\sin (\frac{{J}^{2}}{ {\mathcal E} }t)\,\sin (\frac{{J}^{3}}{2{ {\mathcal E} }^{2}}t),\end{array}$$

which implies that the pseudo-spin polarization vector precesses around the *x*-axis periodically and its module changes with time. By choosing $\alpha =\frac{\pi }{2}$, *β* = 0 to assure the module of the pseudo-spin polarization vector being a constant, we have an *X*-gate for a single qubit. In Fig. 3, we plot the time evolutions of the *y*- and *z*-components of the pseudo-spin polarization vector without considering the fluctuations of parameters. From this figure, we can find that the *y*- and *z*-components of the polarization vector oscillate with time and the phase difference between such two components is $\frac{\pi }{2}$, which exhibits an operating process of an *X*-gate for a single qubit.

**Figure 4:**
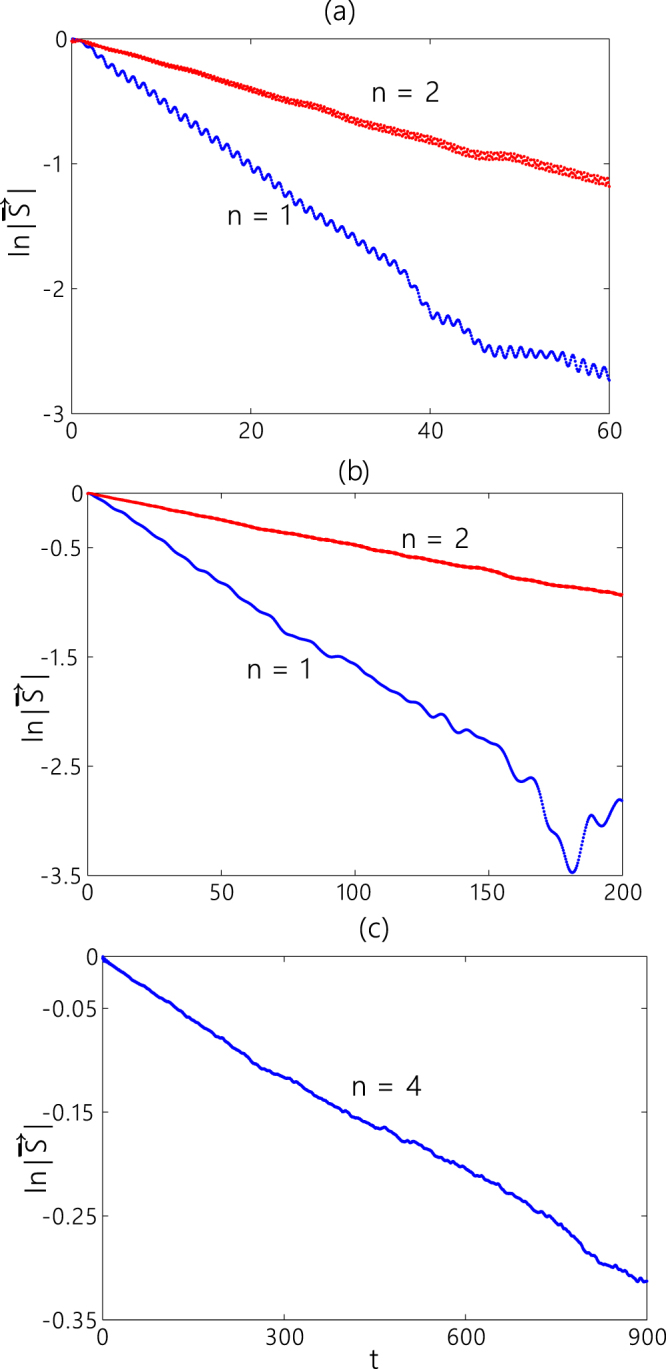
Time evolutions of the logarithm of the length of the pseudo-spin polarization vector for the graph of different sites. The parameters are $\bar{J}=10$ and $\bar{ {\mathcal E} }=50$ in (**a**) and $\bar{ {\mathcal E} }=100$ in (**b**,**c**). The initial states are |1, −〉 for the graph of three sites (*n* = 1), $(|2,-\,\rangle +|1,-\,\rangle )/\sqrt{2}$ for the graph of five sites (*n* = 2), and $|{k}_{-}^{\mathrm{(1)}}\rangle +|{k}_{+}^{\mathrm{(1)}}\rangle$ for the graph of 9 sites (*n* = 4).

Additionally, we also investigate the decoherence effect caused by the fluctuation of the parameters for the five-site graph. The analytical calculation is analogous to that for the previously studied three-site graph. Our result exhibits that the ratio of decoherence for the five-site graph is $\exp [\,-\,{(\bar{J}/\bar{ {\mathcal E} })}^{2}I(t)]$, which implies that the coherence time is four-times longer than that for the three-site graph. In Fig. 4, we plot the time evolution of the logarithm of the length of the polarization vector for the constant spectrum with a cutoff. The curves plotted in Fig. 4 is obtained by numerical method. Figure 4 shows that the module of the polarization vector almost decays exponentially with time when the time scale is not very long, and the coherence time will be enhanced by increasing $\bar{ {\mathcal E} }/\bar{J}$. Comparing the two curves in panels (a) and (b) of Fig. 4, we can find that the decay rate for the graph of three sites is about four times faster than that for the graph of five sites, which is in agreement with our analytical results.

**Figure 5:**
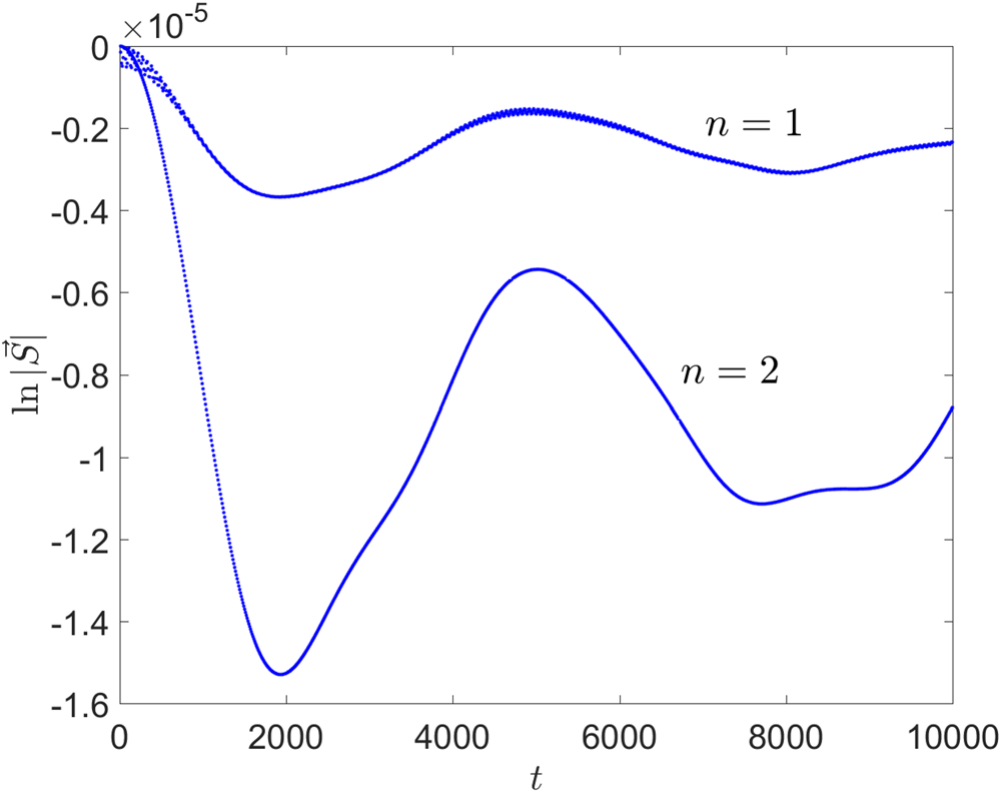
Time evolutions of the logarithm of the length of the pseudo-spin polarization vector for the graph of different sites. The parameters are $\bar{J}=1$ and $\bar{ {\mathcal E} }=1000$. The initial states are |1, −〉 for the graph of three sites (*n* = 1), $(|2,-\,\rangle +|1,-\,\rangle )/\sqrt{2}$ for the graph of five sites (*n* = 2).

Since the quantum dot systems(charge qubit) and the superconductor circuits are usually disturbed by the 1/*f* noise^36,37^, we also consider the decoherence for the noise with 1/|*ω*| spectrum. For the case of 1/|*ω*| spectrum, $I(t)=\frac{1}{\pi }\,{\int }_{{\omega }_{{\rm{l}}}}^{{\omega }_{{\rm{h}}}}\,\frac{{\sin }^{2}\,(\omega t/2)}{{\omega }^{3}\tilde{A}(\omega )}{\rm{d}}\omega$, where *ω*_l_ and *ω*_h_ are lower and higher frequency cutoffs, respectively. The analytical details are presented in the supplementary material. In Fig. 5, we plot the time evolution of the logarithm of the length of the polarization vector for the 1/*f* noise by numerical method. The lower and higher frequency cutoffs of the noise are set to 0.001*J* and 0.003*J*, respectively. We can find that the decay rates oscillate with time for the short time at the beginning, but those tend to constants for long time. We can also find that the decay rate for the graph of three sites is about four times larger than that for the graph of five sites, which confirm our analytical results.

Note that in the above discussion of the decoherence effect, we assume that the noise is ideal, so that each hopping strength between the nearest sites fluctuates synchronously. Whereas, in the real system, the larger system may suffer more complicated noises which can break the synchronization of the hopping strength, then the system will decohere quickly. Note that in the numerical calculation, we have assumed that the frequency of the noise is low enough to guarantee the validity of the adiabatic theory. Besides, the gap between different eigenvalues decreases as increasing chain length. Thus a larger system is influenced by high-frequency noise more seriously. Combining these consideration, for real systems, it may exist an optimal chain length which depends on experimental conditions.

#### The general case

Now we generalize our study to the case of the graph of any odd number of sites since we have constructed an *X*-gate by considering the quantum walk on the one-dimensional graph of three or five sites. As the Hamiltonian (2) is invariant under spatial-inversion, i.e., $l\leftrightarrow -\,l$, the total Hilbert space is decomposed into ${\mathbb{H}}={{\mathbb{H}}}_{+}\oplus {{\mathbb{H}}}_{-}$, where ${{\mathbb{H}}}_{+}$ and ${{\mathbb{H}}}_{-}$ refer to symmetric and antisymmetric Hilbert subspaces, respectively. Then the Hamiltonian (2) is diagonalized as follows, *H*|*k*_−_〉 = 2 cos(*k*_−_) |*k*_−_〉 and *H*|*k*_+_〉 = 2 cos(*k*_+_) |*k*_+_〉 with 〈*l*, +|*k*_−_〉 = sin(*k*_−_*l*), 〈*l*, −|*k*_−_〉 = −〈*l*, +|*k*_−_〉, 〈*l*, −|*k*_+_〉 = 〈*l*, +|*k*_+_〉 = exp(*ik*_+_*l*) + Λ(*k*_+_) exp(−*ik*_+_*l*) and 〈0|*k*_−_〉 = 0, in which ${\rm{\Lambda }}({k}_{+})=(2iJ\,\sin \,{k}_{+}+ {\mathcal E} )/(2iJ\,\sin \,{k}_{+}- {\mathcal E} )$. Here *k*_+_ and *k*_−_ fulfill the following equations,

10 $$\begin{array}{rcl}\sin \,[(n+1){k}_{-}] & = & 0,\\ \frac{2iJ\,\sin \,{k}_{+}- {\mathcal E} }{2iJ\,\sin \,{k}_{+}+ {\mathcal E} } & = & -{e}^{-2i(n+1){k}_{+}}.\end{array}$$

We can solve the real roots from the above secular equation, namely ${k}_{-}^{(m)}=m\pi /(n+1)$, and ${k}_{+}^{(m)}=m\pi /(n+1)$ + $(2J/ {\mathcal E} )\,\sin (\frac{m\pi }{n+1})/(n+1)$ for the case of ${\mathcal E} \gg J$, where *m* = 1, 2, …, *n*. We take the subspace spanned by $|{k}_{+}^{(m)}\rangle$ and $|{k}_{-}^{(m)}\rangle$ for even *m* as the two levels of a qubit. Because $\langle l,-|{k}_{+}^{(m)}\rangle =\langle l,+|{k}_{+}^{(m)}\rangle =\,\sin \,[{k}_{+}^{(m)}(l-n-\mathrm{1)]}$, we can write the reduced density matrix at the initial time as $\rho (0)=1/2+{\hat{\sigma }}_{z}/2$ if the initial state of the system is $|{k}_{+}^{(m)}\rangle +|{k}_{-}^{(m)}\rangle$, where the terms including $J/ {\mathcal E}$ have been neglected due to the fact that ${\mathcal E} \gg J$. Note that the energy difference between the two levels of $|{k}_{+}^{(m)}\rangle$ and $|{k}_{-}^{(m)}\rangle$ is $(4{J}^{2}/ {\mathcal E} )\,{\sin }^{2}(\frac{m\pi }{n+1})/(n+\mathrm{1)}$. Then the effective Hamiltonian for the quantum walks on the graph of 2*n* + 1 sites reads

$${H}_{{\rm{eff}}}^{^{\prime\prime} }=-\,\frac{{J}^{2}}{ {\mathcal E} }\frac{\lambda (m)}{n+1}{\hat{\sigma }}_{x},$$

where $\lambda (m)=2\,{\sin }^{2}(\frac{m\pi }{n+1})$. Additionally, we also consider an adiabatic fluctuation of the parameter *J*, we obtain that the decoherence ratio is $\exp \,[-\frac{4{\lambda }^{2}}{{(n+1)}^{2}}{(\frac{\bar{J}}{\bar{ {\mathcal E} }})}^{2}I(t)]$, which implies that the coherence time can be enhanced up to *n*^2^-times by increasing the length of the chain. We plot the numerical results for the graph of 9 sites (i.e., *n* = 4) in the panel (c) of Fig. 4, which confirms that the increase of the number of sites on the graph can extend the coherence time.

Note that the operate time *τ*_*n*_ of the (2*n* + 1)-point graphs satisfy ${\tau }_{n}\propto n$. For the noises with either a constant spectrum or the Gaussian spectrum or the Lorentz spectrum, the decoherence ratio at time *τ*_*n*_ decrease with *n* in the condition of $I(t)\propto t$. Whereas, for the 1/*f* noise, the conclusion becomes more complicated. For the short operation time (i.e., ${\omega }_{h}{\tau }_{n}\ll 1$), the decoherence ratio at time *τ*_*n*_ has almost nothing to do with the length of the chain because $I(t)\propto {t}^{2}$ for the case of ${\omega }_{h}t\ll 1$. But for the long operation time (i.e., ${\omega }_{h}{\tau }_{1}\gg 1$), the decoherence ratio at time *τ*_*n*_ decrease with *n*^2^ because *I*(*t*) is approximated to a constant for the case of ${\omega }_{l}t\gg 1$.

To understand why increasing length of the chain can protect qubit from noises, one may regard the chain as a auxiliary system, where total Hilbert space has composition ${{\mathbb{H}}}_{1}\otimes {{\mathbb{H}}}_{2}\oplus {{\mathbb{H}}}_{3}$, i.e., ${{\mathbb{H}}}_{2}$ spanned by {|+〉, |−〉} is the Hilbert space of qubit system and ${{\mathbb{H}}}_{1}$ spanned by {|*l*〉 |*l* = 1, 2, …, *n*} is the space of auxiliary system. Then increasing length of the chain is essentially increasing the size (dimension of Hilbert space) of the auxiliary system, which can protect the qubit from noises.

### The construction of control gates for two qubits

In order to construct a control gate for two qubits, we consider a single-particle quantum walk on the following star graph:

The total Hamiltonian reads

11 $$H=\sum _{\alpha ,\beta }\,\sum _{j=1}^{n-1}\,({J}_{\alpha ,\beta }|\alpha ,\beta ,j\rangle \langle \alpha ,\beta ,j+1|+{J^{\prime} }_{\alpha ,\beta }|\alpha ,\beta ,n\rangle \langle 0|+{\rm{H}}.{\rm{c}}.\,)+ {\mathcal E} |0\rangle \langle 0|,$$

where *α*, *β* = 0, 1 which label the four chains connecting with the central site |0〉, and *J*_*α*,*β*_ and ${J^{\prime} }_{\alpha ,\beta }$ are the hopping strengths between the nearest sites. In the following discussion, we take ${J}_{\alpha ,\beta }=[\frac{3}{2}+\frac{1}{2}{(-1)}^{\alpha }]\,J$, ${J^{\prime} }_{\alpha ,\beta }=J\sqrt{\frac{3}{2}+\frac{1}{2}{(-1)}^{\alpha }}$, and show that for such hopping strengths, the quantum-walk system on the above star graph can be used to construct a control gate for two qubits.

Just like the aforementioned case of one-dimensional chain, the eigenvectors |*ψ*〉 for Hamiltonian (11) can be written as

12 $$|\psi \rangle =\sum _{\alpha ,\beta }\,\sum _{j=1}^{n}\,{A}_{\alpha ,\beta }\,\sin ({k}_{\alpha ,\beta }j)|\alpha ,\beta ,j\rangle +{C}_{0}|0\rangle ,$$

where *k*_0,0_ = *k*_0,1_ = *k*_1_, *k*_1,0_ = *k*_1,1_ = *k*_2_, *A*_*α*,*β*_ and *C*_0_ are coefficients to be determined. Substituting Eq. (12) into the Schrödinger equation

13 $$H|\psi \rangle =E|\psi \rangle ,$$

where *E* = 4*J* cos(*k*_1_) = 2*J* cos(*k*_2_) is the eigenvalue, we can determine the coefficients by the following equation

14 $$(\begin{array}{ccccc}\sin \,[{k}_{1}(n+1)] & 0 & 0 & 0 & -\frac{\sqrt{2}}{2}\\ 0 & \sin \,[{k}_{1}(n+1)] & 0 & 0 & -\frac{\sqrt{2}}{2}\\ 0 & 0 & \sin \,[{k}_{2}(n+1)] & 0 & -1\\ 0 & 0 & 0 & \sin \,[{k}_{2}(n+1)] & -1\\ \sqrt{2}\,\sin ({k}_{1}n) & \sqrt{2}\,\sin ({k}_{1}n) & \sin ({k}_{2}n) & \sin ({k}_{2}n) & \frac{ {\mathcal E} -E}{J}\end{array})(\begin{array}{c}{A}_{0,0}\\ {A}_{0,1}\\ {A}_{1,0}\\ {A}_{1,1}\\ {C}_{0}\end{array})=M(\begin{array}{c}{A}_{0,0}\\ {A}_{0,1}\\ {A}_{1,0}\\ {A}_{1,1}\\ {C}_{0}\end{array})=0.$$

Then we obtain the secular equation det*M* = 0, i.e.,

15 $$\begin{array}{l}\sin \,[{k}_{1}(n+1)]\,\sin \,[{k}_{2}(n+1)]\{\frac{ {\mathcal E} -E}{J}\,\sin \,[{k}_{1}(n+1)]\,\sin \,[{k}_{2}(n+1)]\\ \begin{array}{rcl}\,\,\,+2\,\sin ({k}_{1}n)\,\sin \,[{k}_{2}(n+1)]+2\,\sin \,[{k}_{1}(n+1)]\,\sin ({k}_{2}n)\} & = & 0.\end{array}\end{array}$$

Two series of exact solutions for the above equation can be obtained by the equation

16 $$\sin \,[{k}_{1}(n+1)]\,\sin \,[{k}_{2}(n+1)]=0.$$

For the condition that *n* + 1 is even, one solution of the above equation is *k*_1_ = *k*_2_ = *π*/2. One can find that such a solution gives three-fold degeneracy of eigenvalue which can’t be used to construct a control gate for two qubits. Thus in the later discussion, we neglect this solution and focus on the other solutions. For the other solutions we write them in explicit form

17 $$\begin{array}{c}|{\psi }_{0}^{m}\rangle =\sqrt{\frac{1}{n+1}}\,\sum _{j}\,(\sin (\frac{mj\pi }{n+1})|0,0,j\rangle -\,\sin (\frac{mj\pi }{n+1})|0,1,j\rangle ),\\ |{\psi }_{1}^{m^{\prime} }\rangle =\sqrt{\frac{1}{n+1}}\,\sum _{j}\,(\sin (\frac{m^{\prime} j\pi }{n+1})|1,0,j\rangle -\,\sin (\frac{m^{\prime} j\pi }{n+1})|1,1,j\rangle ).\end{array}$$

In the above equations, *m*, *m*′ = 1, 2, 3, …, *n*. The eigenvalues for $|{\psi }_{0}^{m}\rangle$ and $|{\psi }_{1}^{m^{\prime} }\rangle$ are ${E}_{0}^{m}=4J\,\cos (\frac{m\pi }{n+1})$ and ${E}_{1}^{m^{\prime} }=2J\,\cos (\frac{m\pi }{n+1})$, respectively. The other solutions can be obtained by the equation

18 $$\frac{ {\mathcal E} -E}{J}\,\sin \,[{k}_{1}(n+1)]\,\sin \,[{k}_{2}(n+1)]+2\,\sin ({k}_{1}n)\,\sin \,[{k}_{2}(n+1)]+2\,\sin \,[{k}_{1}(n+1)]\,\sin ({k}_{2}n)=0.$$

Since we are interested in the regime ${\mathcal E} /J\gg 1$, the real solutions for Eq. (18) can be solved by sin[*k*_1_(*n* + 1)] sin[*k*_2_(*n* + 1)] ≈ 0. We obtain two series of solutions which can be written as

$$\begin{array}{ll}\{\begin{array}{l}{k}_{1}^{m}=\frac{m\pi }{n+1}+{\delta }_{1},\\ {k}_{2}^{m}=\arccos [2\,\cos ({k}_{1}^{m})],\end{array} & \{\begin{array}{l}{k}_{2}^{m^{\prime} }=\frac{m^{\prime} \pi }{n+1}+{\delta }_{2},\\ {k}_{1}^{m^{\prime} }=\arccos [\frac{1}{2}\,\cos ({k}_{2}^{m^{\prime} })],\end{array}\end{array}$$

where *m*, *m*′ = 1, 2, 3, …, *n*. Taking the first-order approximation of Eq. (18), we obtain $\delta ={\delta }_{1}={\delta }_{2}=\,\sin (\frac{m\pi }{n+1})\times \frac{2}{n+1}\frac{J}{ {\mathcal E} }$. Then we have ${\tilde{E}}_{0}^{m}\approx 4J\,\cos (\frac{m\pi }{n+1})-8J\delta \,\sin (\frac{m\pi }{n+1})$ and ${\tilde{E}}_{1}^{m}\approx 2J\,\cos (\frac{m\pi }{n+1})-4J\delta \,\sin (\frac{m\pi }{n+1})$. In the first-order approximation, the eigenvectors are written as

$$\begin{array}{c}|{\varphi }_{0}^{m}\rangle \approx \sqrt{\frac{1}{n+1}}\,\sum _{j}\,(\sin (\frac{mj\pi }{n+1})|0,0,j\rangle +\,\sin (\frac{mj\pi }{n+1})|0,1,j\rangle ),\\ |{\varphi }_{1}^{m^{\prime} }\rangle \approx \sqrt{\frac{1}{n+1}}\,\sum _{j}\,(\sin (\frac{m^{\prime} j\pi }{n+1})|1,0,j\rangle +\,\sin (\frac{m^{\prime} j\pi }{n+1})|1,1,j\rangle ),\end{array}$$

where the eigenvalue corresponding to $|{\varphi }_{0}^{m}\rangle$, $|{\varphi }_{1}^{m}\rangle$ are ${\tilde{E}}_{0}^{m}$ and ${\tilde{E}}_{1}^{m}$, respectively.

We choose $|{\psi }_{0}^{m}\rangle$, $|{\varphi }_{0}^{m}\rangle$, $|{\psi }_{1}^{m}\rangle$ and $|{\varphi }_{1}^{m}\rangle$ as the bases states for the control gate. Rewrite $\sqrt{\frac{2}{n+1}}\,{\sum }_{j}\,\sin (\frac{mj\pi }{n+1})|\alpha ,\beta ,j\rangle$ by $|\alpha \rangle \otimes |\beta \rangle$, and these four eigenvectors are expressed as

$$\begin{array}{rcl}|{\psi }_{0}^{m}\rangle  & = & \frac{1}{\sqrt{2}}(|0\rangle \otimes |0\rangle -|0\rangle \otimes |1\rangle ),\\ |{\psi }_{1}^{m}\rangle  & = & \frac{1}{\sqrt{2}}(|1\rangle \otimes |0\rangle -|1\rangle \otimes |1\rangle ),\\ |{\varphi }_{0}^{m}\rangle  & = & \frac{1}{\sqrt{2}}(|0\rangle \otimes |0\rangle +|0\rangle \otimes |1\rangle ),\\ |{\varphi }_{1}^{m}\rangle  & = & \frac{1}{\sqrt{2}}(|1\rangle \otimes |0\rangle +|1\rangle \otimes |1\rangle ).\end{array}$$

On the basis of these four eigenvectors, the effective Hamiltonian becomes

19 $${H}_{{\rm{eff}}}^{{\rm{c}}}=({a}_{m}-{{\rm{\Delta }}}_{m}){\hat{\sigma }}_{z}\otimes \hat{I}-3{{\rm{\Delta }}}_{m}\hat{I}\otimes {\hat{\sigma }}_{x}-{{\rm{\Delta }}}_{m}{\hat{\sigma }}_{z}\otimes {\hat{\sigma }}_{x},$$

where ${\hat{\sigma }}_{x,y,z}$ are Pauli matrices and $\hat{I}$ is the two-dimensional identity operator, ${a}_{m}=J\,\cos (\frac{m\pi }{n+1})$ and ${{\rm{\Delta }}}_{m}=\frac{{J}^{2}}{(n+1) {\mathcal E} }\,{\sin }^{2}(\frac{m\pi }{n+1})$. Here we have neglected the constant term in the effective Hamiltonian. Then we obtain the effective time-evolution operator $\hat{U}(t)=\exp (\,-\,i{H}_{{\rm{eff}}}^{{\rm{c}}}t)$. If we consider an operation time $t=\frac{\pi }{4{{\rm{\Delta }}}_{m}}$, the time-evolution operator becomes

20 $$\hat{U}(\frac{\pi }{4{{\rm{\Delta }}}_{m}})=\exp (i\frac{3\pi }{2})\,\exp (i{\theta }_{m}{\hat{\sigma }}_{z}\otimes \hat{I})\,(|0\rangle \langle 0|\otimes \hat{I}+|1\rangle \langle 1|\otimes {\hat{\sigma }}_{x}),$$

where ${\theta }_{m}=\frac{{a}_{m}\pi }{4{{\rm{\Delta }}}_{m}}+\frac{\pi }{4}$. From the above expression, we can find that the system constructs a control gate for two qubits, which is product of a control-NOT gate and a single-qubit phase gate.

Now, we are in the position to discuss the decoherence of such system. We consider the random fluctuation of parameters *J* and ${\mathcal E}$ which can directly lead to fluctuation of Δ_*m*_ and *a*_*m*_. One can find that the decoherence caused by fluctuation of Δ_*m*_ can be suppressed via increasing the chain’s length *n* efficiently, but the fluctuation of *a*_*m*_ still breaks coherence significantly. This is opposite to the case of X-gate, because the term of *a*_*m*_ does not appear in the effective Hamiltonian for X-gate. We will show that choosing appropriate *m*, i.e. $\frac{m\pi }{n+1}\approx \pi /2$ and increasing *n* simultaneously can protect the control gate from the noise induced by the fluctuation of *a*_*m*_ efficiently. Considering a fluctuation of $\delta {a}_{m}=\delta J(t)\,\cos (\frac{m\pi }{n+1})$, we calculate the decoherence ratio that is proportional to $\exp [-{\cos }^{2}(\frac{m\pi }{n+1})I(t)]$. In the condition of $n\gg 1$ and $\frac{m\pi }{n+1}=\pi /2-\frac{\pi }{n+1}$, the decoherence ratio approximates to ${(\frac{\pi }{n+1})}^{2}$ which implies that the coherence time of the constructed gate increases with *n*^2^. Meanwhile, in such a condition, the operation time $t=\frac{\pi }{4{{\rm{\Delta }}}_{m}}\propto n$, so we can find that the decoherence ratio to the operation time increases linearly with the increasing the length of the chain. Note that ${\theta }_{m}=\frac{{\pi }^{2} {\mathcal E} }{2J}+\frac{\pi }{4}+O(\frac{1}{n+1})$ is almost independent of the number of sites on the chain if *n* is large enough.

In the numerical simulation for the control gate, we consider the Gaussian noise which is the same as the case for the X-gate. We calculate the ensemble average of the density matrix $\bar{\rho }={\langle \rho (\frac{\pi }{4{{\rm{\Delta }}}_{m}})\rangle }_{{\rm{env}}}$ at time $t=\frac{\pi }{4{{\rm{\Delta }}}_{m}}$, where *n* is even and $m=\frac{n}{2}$. Then we obtain the coherence ratio ${\rm{Tr}}({\bar{\rho }}^{2})$. The results are presented in Tables 1 and 2. The numerical results indicate that the coherence ratio becomes larger with the increasing of the length of the chain, which confirms our previous prediction.

**Table 1:** The relation between the coherence ratio and the chain length *n* for the constructed control gate.

n	${\bf{T}}{\bf{r}}({\bar{{\boldsymbol{\rho }}}}^{{\bf{2}}})$
2	0.5230
8	0.6680
10	0.7149
16	0.8021
20	0.8422

The spectrum of the noise is constant.

**Table 2:** The relation between the coherence ratio and the chain length *n* for the constructed control gate.

n	${\bf{T}}{\bf{r}}({\bar{{\boldsymbol{\rho }}}}^{{\bf{2}}})$
2	0.5898
4	0.7725
6	0.9401
8	0.9730

The spectrum of the noise is 1/|*ω*|. $\bar{ {\mathcal E} }=10000$, *J* = 1. *ω*_l_ and *ω*_h_ are set to 0.001 and 0.003, respectively.

## Conclusion and Discussion

In this paper, we have investigated the dynamical properties of the continuous-time quantum walk of a particle in a one-dimensional graph of odd number of sites where the on-site potential at the central site differs from that at the other sites. In order to make a more clear physical picture, we gave our analyses on the cases for the graph of three sites and five sites successively, and then presented our result for the general case of the graph containing odd number of site. We showed that the quantum-walk system can construct an *X*-gate for a single qubit when the on-site potential at the central site is much larger than the hopping strength between the nearest sites. We also investigated the decoherence effect of the system by introducing fluctuations to the parameters of the system. We found that the coherence time of the system can be enhanced by increasing either the number of sites on the graph or the ratio of the parameter ${\mathcal E}$ to *J*. This is expected to motivate the design of the quantum gate with long coherence time. We also suggested two experimental proposals to realize the model we considered. One proposal is to connect two coupled superconductor LC circuits via a microwave resonator. Another proposal is a quantum dot array with different voltage at the central site. Additionally, we proposed a quantum-walk system which can be employed to construct a control gate for two qubits. We showed that the coherence time of the constructed control gate can be enhanced by increasing the number of sites on the chain and choosing appropriate eigenvector.

Here we used a single-particle quantum walk to construct quantum gates, which is different from the cases of refs^22,24^ where multiparticles were introduced in those systems. We considered a continuous-time quantum walk on a finite graph and utilized the qusi-momentum eigenstates of the quantum-walk system in the process of constructing the quantum gates. Whereas, the graphs considered in refs^2,21^ is infinite although the scattering sources are finite. In our scheme, we introduced the auxiliary states to protect the qubit systems, which differs from the case of ref.^23^.

## Methods

In numerical calculation of the decoherence of the quantum gate, we use Gaussian random number generator to simulate the constant-spectrum noise and set Δ = 3. And some details of mathematical analysis is presented in Appendix.

## Electronic supplementary material

Appendix
