# Supplementary material for: A Rout to Protect Quantum Gates constructed via quantum walks from Noises
Source: Sci Rep. 2018 May 8;8:7117. doi: 10.1038/s41598-018-25550-1 (PMC5940792; doi:10.1038/s41598-018-25550-1)
Supplement: Supplementary file 1 — Appendix [file 41598_2018_25550_MOESM1_ESM.pdf]

A Rout to Protect Quantum Gate constructed via quantum walk from Noises

Yi-Mu Du[1*], Li-Hua Lu[1*] and You-Quan Li[1,2]

[1] *Zhejiang Institute of Modern Physics and Department of Physics,*
Zhejiang University, Hangzhou 310027, P. R. China
[2] *Collaborative Innovation Center of Advanced Microstructures, Nanjing, P. R. China*[*]

* lhlu@zju.edu.cn, 11536016@zju.edu.cn

APPENDIX A: DETAILS OF THE EFFECTIVE HAMILTONIAN OF THE COUPLED LC CIRCUITS

With the help of the Kirchhoff's law, the motion equations of particles for the left series of the superconductor LC circuits can be given as

$$(L_1+M)\frac{\mathrm{d}^2q_1}{\mathrm{d}t^2}+\frac{q_1+q_2}{C}=0,$$
$$(L_2+M)\frac{\mathrm{d}^2q_2}{\mathrm{d}t^2}+\frac{q_1+q_2}{C}+Vq_2=0,$$

where $L_{1(2)}$ refers to the self-inductance, M to the mutual-inductance, C to the capacitance, and V to the AC voltage provided by the microwave resonator. According to the above motion equations of particles, we can write the Lagrangian as

$$\mathcal{L}_\mathrm{L}=\frac{L_1}{2}(\frac{\mathrm{d}q_1}{\mathrm{d}t})^2+\frac{L_2}{2}(\frac{\mathrm{d}q_2}{\mathrm{d}t})^2+M\frac{\mathrm{d}q_1}{\mathrm{d}t}\frac{\mathrm{d}q_2}{\mathrm{d}t}-\frac{(q_1+q_2)^2}{2C}-Vq_2.$$

With the help of canonical equation, we can obtain a set of canonical momentum

$$\begin{pmatrix} p_1 \\ p_2 \end{pmatrix}=\begin{pmatrix} L_1 & M \\ M & L_2 \end{pmatrix}\begin{pmatrix} \frac{\mathrm{d}q_1}{\mathrm{d}t} \\ \frac{\mathrm{d}q_2}{\mathrm{d}t} \end{pmatrix}.$$

Then the classical Hamiltonian describing the left series of the superconductor circuits is

$$\mathcal{H}_\mathrm{L}=\frac{p_1^2}{2\mu_1}+\frac{p_2^2}{2\mu_2}+\frac{q_1^2+q_2^2}{2C}+\lambda_1p_1p_2+\lambda_2q_1q_2+Vq_2, \tag{A1}$$

where

$$\mu_1=\frac{L_1L_2-M^2}{L_2},$$
$$\mu_2=\frac{L_1L_2-M^2}{L_1},$$
$$\lambda_1=\frac{M}{L_1L_2-M^2},$$
$$\lambda_2=\frac{1}{C}.$$

In order to quantized the classical Hamiltonian (A1), we introduce two creation and annihilation operators $a_j=\frac{1}{\sqrt{2}}(\frac{\hat{q}_j}{\sqrt{C/\mu_j}}-\mathrm{i}\frac{\hat{p}_j}{\sqrt{\mu_j/C}})$, $a_j^\dagger=\frac{1}{\sqrt{2}}(\frac{\hat{q}_j}{\sqrt{C/\mu_j}}+\mathrm{i}\frac{\hat{p}_j}{\sqrt{\mu_j/C}})$ with $(j=1,2)$ since $\hat{p}_j$ and $\hat{q}_j$ satisfy the canonical communication relation $[\hat{q}_j,\hat{p}_{j'}]=\mathrm{i}\delta_{jj'}$. Then the two-order quantization form of the classical Hamiltonian (A1) is

$$\begin{aligned}\hat{\mathcal{H}}_\mathrm{L}=&(\frac{\lambda_1\sqrt{\mu_1\mu_2}}{2C}+\frac{\lambda_2C}{2\sqrt{\mu_1\mu_2}})(a_1^\dagger a_2+a_1a_2^\dagger)\\&+(\frac{\lambda_1\sqrt{\mu_1\mu_2}}{2C}-\frac{\lambda_2C}{2\sqrt{\mu_1\mu_2}})(a_1^\dagger a_2^\dagger+a_1a_2)\\&+\omega_1a_1^\dagger a_1+\omega_2a_2^\dagger a_2+V\sqrt{\frac{C}{2\mu_2}}(a_2^\dagger+a_2)+\frac{\omega_1+\omega_2}{2},\end{aligned}$$

where $\omega_j=\frac{1}{\sqrt{\mu_jC}}$. If we take the coefficients to satisfy the condition $\mu_1=\mu_2=\mu$ and $\frac{\lambda_1}{\lambda_2}=\frac{C^2}{\mu_1\mu_2}$ through tuning $L_{1(2)}$, M and C, the Hamiltonian of the left series of the superconductor LC circuits can be simplified as

$$\hat{\mathcal{H}}_\mathrm{L}=\omega(a_1^\dagger a_1+a_2^\dagger a_2)+J(a_1^\dagger a_2+a_2^\dagger a_1)+V\sqrt{\frac{C}{2\mu}}(a_2^\dagger+a_2), \tag{A2}$$

where $\omega = \frac{1}{\sqrt{\mu C}}$, $J = \frac{1}{\mu}$ and the constant term is neglected without affecting the physical properties of the system. The Hamiltonian describing the right series of the superconductor LC circuits is in the same form with $\hat{\mathcal{H}}_{\rm L}$ just replacing $a_{1(2)}$ by $a_{3(4)}$, respectively,

$$\hat{\mathcal{H}}_{\rm R} = \omega(a_3^\dagger a_3 + a_4^\dagger a_4) + J(a_4^\dagger a_3 + a_3^\dagger a_4) + V\sqrt{\frac{C}{2\mu}}(a_3^\dagger + a_3). \tag{A3}$$

The microwave resonator bridging the two series of superconductor LC circuits can be quantized just like the LC circuit. For a particular mode $k = 2\pi/d$, the voltage in the microwave resonator in Schödinger picture is

$$\hat{V}(x) = -\sqrt{\frac{\omega_0}{d\tilde{c}}}\sin(kx)(a^\dagger + a),$$

where $\omega_0 = 2\pi/(d\sqrt{L_0 C_0})$ with d referring to the length of the resonator, L_0 and C_0 to the inductance and capacitance per unit length of the resonator, respectively. Then the AC voltage V in Eq. (A2) and Eq. (A3) can be quantized with the help of

$$V = \hat{V}(\frac{3d}{4}) - \hat{V}(\frac{d}{4}).$$

So the coupling term between the resonator and the superconductor LC circuits, i.e., the sum of the third term in Eq. (A2) and Eq. (A3), can be written as

$$\hat{\mathcal{H}}_{\rm int} = \lambda(a^\dagger + a)(a_2^\dagger + a_2 + a_3^\dagger + a_3),$$

where $\lambda = \sqrt{\frac{C\omega_0}{2\mu d C_0}}$. In rotating-wave approximation, the coupling between the resonator and the superconductor LC circuits can be rewritten as

$$\hat{\mathcal{H}}_{\rm int} = \lambda(a^\dagger a_2 + a^\dagger a_3 + \text{H.c}).$$

Taking $\lambda = J$ via tuning the parameter of the system, we have the total Hamiltonian of the superconductor LC circuits bridged by a microwave resonator is

$$\hat{\mathcal{H}} = \omega(a_1^\dagger a_1 + a_2^\dagger a_2 + a_3^\dagger a_3 + a_4^\dagger a_4) + \omega_0 a^\dagger a + J(a_1^\dagger a_2 + a_2^\dagger a + a^\dagger a_3 + a_3^\dagger a_4) + \text{H.c}. \tag{A4}$$

Considering a single-photon process, the Hilbert space of the system describing by Eq. (A4) is spanned by the following vector,

$$\begin{aligned} |-2\rangle &= a_1^\dagger|\text{vac}\rangle, \\ |-1\rangle &= a_2^\dagger|\text{vac}\rangle, \\ |0\rangle &= a^\dagger|\text{vac}\rangle, \\ |1\rangle &= a_3^\dagger|\text{vac}\rangle, \\ |2\rangle &= a_4^\dagger|\text{vac}\rangle, \end{aligned}$$

where $|\text{vac}\rangle$ denotes a vacuum state. Then the Hamiltonian Eq. (A4) can be expressed as

$$H = \sum_{j=-2}^{1} J(|j\rangle\langle j+1| + \text{H.c.}) + \mathcal{E}|0\rangle\langle 0|,$$

where $\mathcal{E} = \omega_0 - \omega$ and the constant term is neglected. That is just the Hamiltonian describing the continuous-time quantum walk of a single particle on the graph of five sites we considered.

APPENDIX B: CALCULATION OF SEVERAL NOISES WITH DIFFERENT SPECTRA

Considering several noises with different spectra, we calculate the corresponding characteristic time of coherence τ and compare it with the operation time τ_0. $\tau_0 = \frac{(n+1)\bar{\mathcal{E}}}{\bar{J}^2}\frac{\pi}{2\lambda(m)}$, which implies that $\tau_0 \propto \kappa$ with $\kappa = \frac{(n+1)\bar{\mathcal{E}}}{\bar{J}}$.

Constant Spectrum

The spectrum of the correlation function is

$$\frac{1}{\tilde{A}(\omega)} = \begin{cases} \Delta^2 & |\omega| \leq \omega_0 \\ 0 & |\omega| > \omega_0 \end{cases}.$$

For the case of large ω_0,

$$\begin{aligned} I(t) &= \frac{1}{2\pi}\int_{-\omega_0}^{+\omega_0} \Delta^2 \frac{\sin^2(\omega t/2)}{\omega^2}\mathrm{d}\omega \\ &\approx \frac{1}{2\pi}\int_{-\infty}^{+\infty} \Delta^2 \frac{\sin^2(\omega t/2)}{\omega^2}\mathrm{d}\omega \\ &= \frac{\Delta^2}{4}t, \end{aligned}$$

which implies that $\tau \propto \kappa^2$. When the ratio $\bar{\mathcal{E}}/\bar{J}$ or the length of the chain increases, τ increases faster than τ_0.

Lorentz Spectrum

The spectrum of the correlation is

$$\tilde{A}(\omega) = \omega^2 + \omega_0^2.$$

The corresponding $I(t)$ is

$$\begin{aligned} I(t) &= \frac{1}{8\pi}\int_{-\infty}^{+\infty} \frac{2 - \mathrm{e}^{\mathrm{i}\omega t} - \mathrm{e}^{-\mathrm{i}\omega t}}{\omega^2(\omega - \mathrm{i}|\omega_0|)(\omega + \mathrm{i}|\omega_0|)}\mathrm{d}\omega \\ &= \frac{1}{4\omega_0^2}(t + \frac{\mathrm{e}^{-\omega_0 t} - 1}{\omega_0}), \end{aligned}$$

which shows that $\tau \propto \kappa^2$.

Gaussian Spectrum

The spectrum of the correlation function is

$$\frac{1}{\tilde{A}(\omega)} = \frac{1}{\sqrt{\pi}}\exp(-\frac{\omega^2}{\sigma^2}).$$

The corresponding $I(t)$ is

$$\begin{aligned} I(t) &= \frac{1}{8\pi}\frac{1}{\sqrt{\pi}}\int_{-\infty}^{+\infty} \frac{2 - \mathrm{e}^{\mathrm{i}\omega t} - \mathrm{e}^{-\mathrm{i}\omega t}}{\omega^2}\frac{1}{\sqrt{\pi}}\exp(-\frac{\omega^2}{\sigma^2})\mathrm{d}\omega \\ &= -\frac{1}{8\pi}\frac{1}{\sqrt{\pi}}\int_{-\infty}^{+\infty}\sum_{k=0}^{\infty}\frac{(-1)^{k+1}t^{2k+2}}{(2k+2)!}\omega^{2k}\exp(-\frac{\omega^2}{\sigma^2})\mathrm{d}\omega \\ &= -\frac{1}{8\pi\sigma}\sum_{k=0}^{\infty}\frac{(-1)^{k+1}(\sigma t)^{2k+2}}{(2k+2)!}\frac{(2k)!}{2^{2k}k!} \\ &= -\frac{1}{4\pi\sigma}\sum_{k=0}^{\infty}\frac{(-1)^{k+1}(\sigma^2 t^2/4)^{k+1}}{(2k+1)(k+1)!}, \end{aligned} \tag{B1}$$

which shows that $I(t)$ monotonically increases with t. Thus we evaluate a characteristic time τ by using following equation

$$I(\tau) = \gamma,$$

where $\gamma = \kappa^2$. From the above equation, we have

$$\frac{\mathrm{d}I}{\mathrm{d}\tau}\frac{\mathrm{d}\tau}{\mathrm{d}\gamma} = 1$$

With the help of Eq. (B1), we have

$$\frac{\mathrm{d}I}{\mathrm{d}t} = \frac{I(t)}{t} - \frac{1}{4\pi\sigma}\frac{\exp(-\sigma^2 t^2/4) - 1}{t}. \tag{B2}$$

Since $I(t)$ is monotonically increasing and $I(0) = 0$, τ is also very large for large γ. In this regime, the equation (B2) can be simplified as

$$\frac{\mathrm{d}I}{\mathrm{d}t} = \frac{I(t)}{t} + \frac{1}{4\pi\sigma}\frac{1}{t}.$$

Then we obtain following relation

$$\frac{1}{\tau}\mathrm{d}\tau = \frac{1}{\gamma + 1/(4\pi\sigma)}\mathrm{d}\gamma,$$

which implies that $\tau(\gamma)$ is proportional to γ. So we have $\tau \propto \kappa^2$.

$1/f$ Spectrum

The spectrum of the correlation function is

$$\frac{1}{\tilde{A}(\omega)} = \frac{1}{|\omega|}.$$

For the given lower and higher cutoffs ω_l, ω_h, we have

$$\begin{aligned} I(t) &= \frac{1}{\pi}\int_{\omega_\mathrm{l}}^{\omega_\mathrm{h}} \frac{\sin^2(\omega t/2)}{\omega^3} \\ &= \frac{1}{\pi}\Big(\int_{\omega_\mathrm{l}}^{\infty} \frac{\sin^2(\omega t/2)}{\omega^3}\mathrm{d}\omega - \int_{\omega_\mathrm{h}}^{\infty} \frac{\sin^2(\omega t/2)}{\omega^3}\mathrm{d}\omega\Big) \\ &= -\frac{1}{\pi}\Big[\frac{1}{\omega_\mathrm{l}^2}f(\omega_\mathrm{l} t) - \frac{1}{\omega_\mathrm{l}^2}f(\omega_\mathrm{h} t)\Big], \end{aligned}$$

where the function $f(t)$ can be expressed as

$$f(t) = -\int_1^{\infty} \frac{\sin^2(\omega t/2)}{\omega^3}\mathrm{d}\omega.$$

The functional image for $f(t)$ is plotted in Fig. 1. One can see from the functional image that $-1 < f(t) < 0$ and $\lim_{t\to+\infty} f(t) = -\frac{1}{4}$.

For the short-time limit $\omega_h t \ll 1$, one can find that $I(t) \propto t^2$. Whereas, for the long-time limit $\omega_\mathrm{l} t \gg 1$, one can see that $I(t) = \frac{1}{4\pi}\big[\frac{1}{\omega_\mathrm{l}^2} - \frac{1}{\omega_\mathrm{l}^2}\big]$. These analytical results imply that increasing the chain's length can protect the system from $1/f$ noises effectively in the case of $\omega_\mathrm{l}\tau_n \gg 1$, where τ_n is operation time.

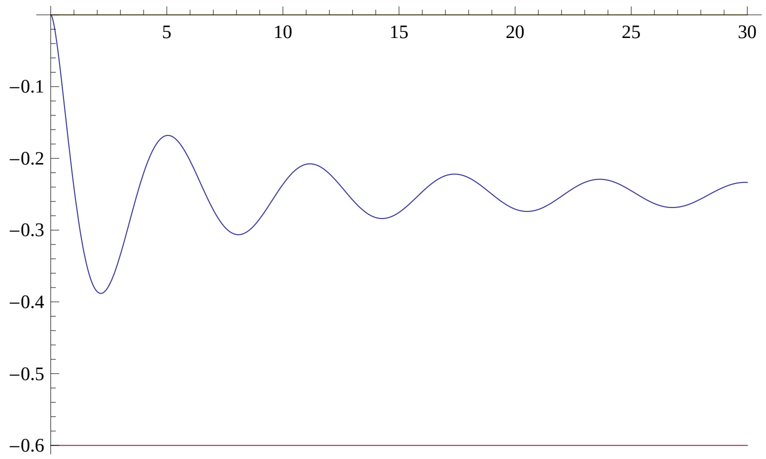

FIG. 1. The functional image for $f(t)$.
